# Discovery of the genus *Formosiepyris* Terayama, (Hymenoptera, Bethylidae) in Vietnam, with a description of a new species

**DOI:** 10.3897/zookeys.507.9773

**Published:** 2015-06-08

**Authors:** Kentaro Tsujii, Toshiharu Mita, Mamoru Terayama, Hong Thai Pham, Shûji Okajima

**Affiliations:** 1 Laboratory of Entomology, Faculty of Agriculture, Tokyo University of Agriculture, 1737 Funako, Atsugi, Kanagawa 243–0034 Japan; 2 Entomological Laboratory, Faculty of Agriculture, Kyushu University, 6-10-1 Hakozaki, Higashi-ku, Fukuoka, 812-8581, Japan; 3 Graduate School of Agricultural and Life Sciences, The University of Tokyo, 1-1-1, Yayoi, Bunkyo-ku, Tokyo, 113-8657, Japan; 4 Vietnam National Museum of Nature, Vietnam Academy of Science and Technology (VAST) 18 Hoang Quoc Viet St, Hanoi, Vietnam

**Keywords:** Epyrinae, key, new record, Oriental region

## Abstract

*Formosiepyris vietnamensis* **sp. n.** (Hymenoptera: Bethylidae) is described based on material collected from Da Lat, southern Vietnam. This is the first record of *Formosiepyris* Terayama from Vietnam. The new species can be distinguished from other *Formosiepyris* species by a narrow and rounded clypeus; a mandible with three teeth; a second metasomal tergite having small, sparsely distributed punctures and smooth interspaces, except for anterior 2/5, which is microreticulate; and a head length : width aspect ratio of 10 : 11. A key to the Oriental species of *Formosiepyris* is provided.

## Introduction

The genus *Formosiepyris* Terayama, 2004 (Hymenoptera: Bethylidae: Epyrinae) was initially described based on three species, namely, *Formosiepyris marishi* Terayama, 2004 from Thailand, *Formosiepyris shiva* Terayama, 2004 from India and *Formosiepyris takasago* Terayama, 2004 from Taiwan (Terayama 2004). Subsequently, *Formosiepyris rugulosus* Xu & He, 2005 was described from Fujian Province, China, (Xu and He 2005). While all of these species are distributed in the Oriental region, Mugrabi and Azevedo (2010) and Azevedo et al. (2010) recorded several unidentified species of *Formosiepyris* in the United Arab Emirates and Madagascar, implying that the distribution and diversity of this genus require clarification.

In the course of our study on the hymenopteran fauna of Vietnam, we collected a male *Formosiepyris* specimen from Da Lat, Lam Dong Province, in southern Vietnam. Here we describe a new *Formosiepyris* species based on this male, and provide an updated key to the Oriental species.

## Material and methods

The terminology follows that of Evans (1964), Azevedo (2001), and Terayama (2006). The following abbreviations were used in the description: HL, head length; HW, head width; WF, width of frons; LM, length of mesosoma; LPD, length of propodeal disc; WPD, width of propodeal disc; FWL, forewing length; TL, total body length; EL, eye length; POL, minimum distance between posterior ocelli; AOL, minimum distance from a posterior ocellus to nearest eye margin; OOL, minimum distance from a posterior ocellus to nearest eye margin; WOT, distance across and including posterior ocelli.

The holotype of *Formosiepyris takasago* deposited in the National Museum of Nature and Science Tsukuba, Japan, and a paratype of *Formosiepyris marishi* in the Terayama Collection were also examined for comparison. Character evaluations of the other two species, *Formosiepyris shiva* and *Formosiepyris rugulosus*, were based on the original descriptions (Terayama 2004; Xu and He 2005).

The holotype designated in this study has temporarily been deposited in the Laboratory of Entomology, Faculty of Agriculture, Tokyo University of Agriculture Atsugi, Japan, but it will be transferred to the Vietnam National Museum of Nature, Hanoi, Vietnam.

## Taxonomy

### Genus Formosiepyris

Taxon classificationAnimaliaHymenopteraBethylidae

Terayama, 2004

Formosiepyris Terayama, 2004: 91. Type species: *Formosiepyris marishi* Terayama, 2004.

#### Diagnosis.

Head rounded; mandible with three or four teeth; median lobe of clypeus rounded or triangular; lateral lobe of clypeus undeveloped; eye covered with minute setae, large and convex; antenna with 13 antennomeres; posterior margin of pronotal disc and mesoscutum with transverse groove; posterolateral corner of propodeal disc with two pairs of small projections (Terayama 2004).

#### Distribution.

Oriental region and Afrotropical region (Species listed in the latter are not identified) (Terayama 2004; Xu and He 2005; Mugrabi and Azevedo 2010; Azevedo et al. 2010).

#### Host.

Unknown (Terayama 2004; Xu and He 2005).

### Formosiepyris vietnamensis

Taxon classificationAnimaliaHymenopteraBethylidae

Tsujii, Mita, Terayama, Pham & Okajima sp. n.

http://zoobank.org/BDE59050-88E1-4EC3-93C0-556D41100590

Figs 1–5

#### Type.

Holotype: male, Vietnam, Lam Dong Prov., Da Lat, near Tuyen Lam Lake, 11°53'00.1"N, 108°24'29.9"E, ca. 1420m, 15. III. 2013, collected by yellow pan trap, K. Tsujii leg.

#### Diagnosis.

**Figures 1–5.**
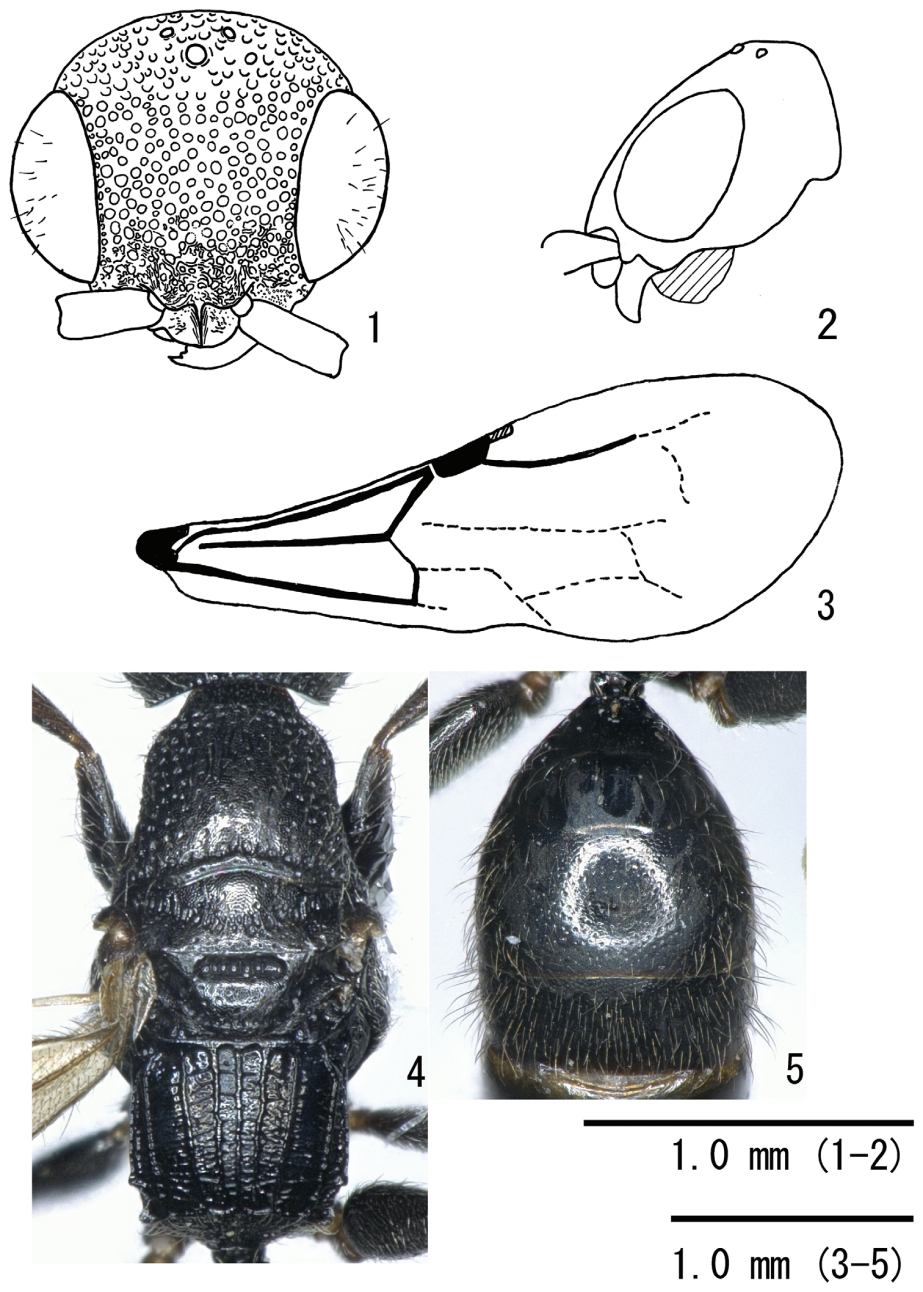
*Formosiepyris vietnamensis* sp. n., holotype male. **1** Head in full face view **2** head in lateral view **3** forewing in dorsal view **4** mesosoma in dorsal view **5** metasoma in dorsal view.

Mandible with three teeth; anterior margin of clypeus rounded (Fig. 1); surface between median carina and inner submedian carina of propodeum without longitudinal striae; second metasomal tergite with sparse small punctures, and with smooth interspaces except anterior 2/5 sparsely microreticulate (Fig. 5).

#### Description of male.

Measurements. HL 0.99 mm; HW 1.10 mm; WF 0.58 mm; LM 1.96 mm; LPD; 0.65 mm; WPD, 0.78 mm; FWL 2.78 mm; TL 4.90 mm. EL 0.56 mm; Malar space 0.89 mm. Hind wing 2.08 mm. Hind leg 2.68 mm.

Coloration. Body black, except dark brown apical half of mandible and yellowish brown tarsi. Wings faintly tinged with brown.

Head. Head 0.9 times as long as wide, with convex posterior margin in frontal view; frons and vertex densely and strongly punctated and microreticulate interspaces; maximum diameter of punctures ca. 0.3–0.5 mm. Posteroventral corner of gena forming dully projection (Fig. 2). Mandible with three teeth (Fig. 1). Anterior margin of clypeus rounded. The ratio of first five antennomeres as follows; 16: 7: 11: 11: 11. WF 1.05 times EL. Ocelli forming obtuse triangle; OOL 1.24 times WOT.

Mesosoma (Fig. 4). Pronotum punctated with microreticulate interspaces; punctures sparser than head; median longitudinal carina absent. Scutum punctated with microreticulate interspaces. Propodeal disc 0.83 times as long as wide, with five discal carinae; surfaces between carinae of propodeum without longitudinal striae; posterolateral corner of propodeal disc with two pairs of distinct projection; sublateral carina present. Hind femur 5.6 times as long as wide in lateral view. Hind leg with two tibial spurs as long as half of first tarsomere. Tarsal claws simple. Fore wing with short metacarpus, 0.4 times as long as pterostigma; transverse-median vein convex posteriorly.

Metasoma. First tergite 0.5 times as long as wide, smooth; second tergite 0.5 times as long as wide; second and third tergites punctated with sparsely located small punctures and smooth interspaces except microreticulated anterior 2/5 (Fig. 5).

Female. Unknown.

#### Etymology.

The specific name is from the type locality, Vietnam.

#### Hosts.

Unknown.

#### Distribution.

Oriental region: Vietnam (Lam Dong Province).

#### Remarks.

This species is similar to *Formosiepyris marishi* based on the convex posterior margin of the head in full-face view; having a punctated pronotum with microreticulate interspaces; punctures on pronotum sparser than those on head; and absence of longitudinal striae on propodeum surface between median carina and inner submedian carina. However, this species can be distinguished from *Formosiepyris marishi* by having a weakly rounded anterior margin of the clypeus; head wider than long; ocelli forming an obtuse triangle; second antennomere shorter than third antennomere; and second and third tergites with small sparsely located punctures and smooth interspaces, except for microreticulated anterior 2/5.

### Key to species of Oriental *Formosiepyris*

Female (females of *Formosiepyris takasago* and *Formosiepyris vietnamensis* are unknown)

**Table:** 

1	Anterior margin of clypeus triangular	***Formosiepyris marishi* Terayama, 2004**
–	Anterior margin of clypeus rounded	**2**
2	Mandible with three teeth; posterior margin of head convex in full-face view; second antennomere as long as third antennomere; surface between median carina and inner submedian carina of propodeum with longitudinal striae	***Formosiepyris shiva* Terayama, 2004**
–	Mandible with four teeth; posterior margin of head straight in full-face view; second antennomere shorter than third antennomere; surface between median carina and inner submedian carina of propodeum without longitudinal striae	***Formosiepyris rugulosus* Xu & He, 2005**

Male (males of *Formosiepyris shiva* and *Formosiepyris rugulosus* are unknown)

**Table:** 

1	Anterior margin of clypeus triangular; ocelli forming a right triangle in full-face view	***Formosiepyris marishi* Terayama, 2004**
–	Anterior margin of clypeus rounded; ocelli forming an obtuse triangle in full-face view	**2**
2	Head as long as wide, with posterior margin straight in full-face view; second metasomal tergite densely punctate	***Formosiepyris takasago* Terayama, 2004**
–	Head wider than long, with posterior margin convex in full-face view (Fig. 1); second metasomal tergite sparsely punctated except anterior 2/5 covered with microreticulate (Fig. 5)	***Formosiepyris vietnamensis* sp. n.**

## Supplementary Material

XML Treatment for Formosiepyris

XML Treatment for Formosiepyris vietnamensis
